# The potential impact of routine testing of individuals with HIV indicator diseases in order to prevent late HIV diagnosis

**DOI:** 10.1186/1471-2334-13-473

**Published:** 2013-10-10

**Authors:** Paola Scognamiglio, Giacomina Chiaradia, Gabriella De Carli, Massimo Giuliani, Claudio Maria Mastroianni, Stefano Aviani Barbacci, Anna Rita Buonomini, Susanna Grisetti, Alessandro Sampaolesi, Angela Corpolongo, Nicoletta Orchi, Vincenzo Puro, Giuseppe Ippolito, Enrico Girardi

**Affiliations:** 1 National Institute for Infectious Diseases “L. Spallanzani” (IRCCS), Via Portuense 292, Rome 00149, Italy; 2 San Gallicano Dermatological Institute (IRCCS), Rome, Italy; 3 CRAIDS S.M. Goretti Hospital, Sapienza University, Latina, Italy; 4 CRAIDS Belcolle Hospital, Viterbo, Italy; 5 Clinic of Infectious Diseases, Tor Vergata University, Rome, Italy

**Keywords:** HIV testing, Indicator diseases, Sexually transmitted infections, Late diagnosis

## Abstract

**Background:**

The aim of our work was to evaluate the potential impact of the European policy of testing for HIV all individuals presenting with an indicator disease, to prevent late diagnosis of HIV. We report on a retrospective analysis among individuals diagnosed with HIV to assess whether a history of certain diseases prior to HIV diagnosis was associated with the chance of presenting late for care, and to estimate the proportion of individuals presenting late who could have been diagnosed earlier if tested when the indicator disease was diagnosed.

**Methods:**

We studied a large cohort of individuals newly diagnosed with HIV infection in 13 counselling and testing sites in the Lazio Region, Italy (01/01/2004-30/04/2009). Considered indicator diseases were: viral hepatitis infection (HBV/HCV), sexually transmitted infections, seborrhoeic dermatitis and tuberculosis. Logistic regression analysis was performed to estimate association of occurrence of at least one indicator disease with late HIV diagnosis.

**Results:**

In our analysis, the prevalence of late HIV diagnosis was 51.3% (890/1735). Individuals reporting at least one indicator disease before HIV diagnosis (29% of the study population) had a lower risk of late diagnosis (OR = 0.7; 95%CI: 0.5-0.8) compared to those who did not report a previous indicator disease. 52/890 (5.8%) late presenters were probably already infected at the time the indicator disease was diagnosed, a median of 22.6 months before HIV diagnosis.

**Conclusions:**

Our data suggest that testing for HIV following diagnosis of an indicator disease significantly decreases the probability of late HIV diagnosis. Moreover, for 5.5% of late HIV presenters, diagnosis could have been anticipated if they had been tested when an HIV indicator disease was diagnosed.

However, this strategy for enhancing early HIV diagnosis needs to be complemented by client-centred interventions that aim to increase awareness in people who do not perceive themselves as being at risk for HIV.

## Background

Combination antiretroviral therapy (cART) has dramatically changed the natural history of HIV infection by substantially reducing associated morbidity and mortality [1]. However, the effectiveness of this treatment at both the individual and population level is limited by the fact that a substantial proportion of persons living with HIV are unaware of their serostatus, and present for clinical care when already at an advanced stage of infection. In Europe, almost 30% of HIV-infected persons still remain undiagnosed [2] and the overall incidence of late presentation may be as high as 50% of all HIV cases [3,4]. In the United States (US), it is estimated that nearly 1.1 million people are HIV-infected but approximately 25% of them are not aware of their infection [5]; in 2007, 54% of persons with HIV who entered into care had a CD4 cell count below the threshold mentioned in different guidelines for cART initiation [6].

Different strategies have been proposed to address this problem. In 2006, the US Centres for Disease Control and Prevention (CDC) recommended routine HIV testing for all individuals aged 13 to 64 years who come into contact with the health system [7]. However, available evidence suggests that uptake of routine HIV screening varies greatly in different health care settings [8]. Some studies showed that although the level of patient acceptability is high (>90%), the test offering rate may be rather low due to staff-generated barriers, including attitudinal barriers (patients are not perceived to be at risk and therefore testing is not offered) [9,10].

In Europe another approach has been proposed, which is based on routine testing of individuals presenting with an “HIV indicator disease”, independent of any risk assessment. These diseases include certain infections that share a common mode of transmission with HIV, diseases whose onset is favoured by HIV-induced immunodeficiency, and any other medical condition associated with an undiagnosed HIV prevalence greater than 0.1%, the prevalence value for which delivering routine HIV testing resulted to be cost-effective [11,12].

The main objective of this study was to assess retrospectively the potential impact of this policy on preventing late HIV diagnosis in a cohort of newly HIV-diagnosed individuals. In particular, we estimated the association of HIV indicator disease occurrence with late HIV diagnosis, identifying variables associated with HIV testing after the diagnosis of an indicator disease and estimating the proportion of late presenting individuals who could have been diagnosed earlier if tested when the indicator disease was diagnosed.

## Methods

Since January 2004, a prospective multi-centre observational study on newly diagnosed adults with HIV infection (SENDIH Study) has been conducted in 13 regional counselling and testing sites of the Lazio region. Characteristics and methods of the study have been previously described [13,14]. Briefly, for all enrolled individuals, the following information was collected in a standardized case report form (see Additional file 1): socio-demographic characteristics, laboratory data, HIV exposure category, previous HIV tests and clinical history.

In particular, based on the frequencies observed in the pilot study, the questionnaire collected information about the following HIV indicator diseases: hepatitis B virus (HBV) and hepatitis C virus (HCV) infection, syphilis, gonorrhoea, genital herpes, genital warts, infectious vaginitis, seborrhoeic dermatitis and tuberculosis.

Regarding tuberculosis, even if it is already a generally accepted practice to screen TB patients for HIV, often, this recommendation is not followed. In addition, a number of studies demonstrated that TB represents a missed opportunity for HIV infection diagnosis [15,16].

The ethics committee of the coordinating centre, the “L. Spallanzani” National Institute for Infectious Diseases has approved the study [13].

In the analysis, we included all individuals enrolled up to April 30, 2009 with a CD4 count determination available within 3 months of HIV diagnosis.

HIV exposure category was classified according to the following modes of acquisition: intravenous drug use (IDU); men who have sex with men (MSM); heterosexuals. Individuals infected through blood products and those without a defined HIV exposure risk factor were classified as “other/unreported”.

In a subset of individuals who also completed a self-administered behavioural and clinical questionnaire, we measured the concordance between the information on each single indicator disease reported in the questionnaire and the data collected at enrolment by Cohen's kappa statistic (K coefficient).

For patients who reported a previous HBV or HCV infection at enrolment, we also checked serological status in clinical and laboratory records and measured the agreement between reported information and serological data.

### Statistical analysis

A descriptive analysis was performed to describe the occurrence of an indicator disease before HIV diagnosis.

Univariable and multivariable logistic regression analysis were performed to estimate the association of HIV indicator diseases with late diagnosis, categorized as “late presentation” or “presentation with advanced HIV disease”. As a measure of association, we calculated the odds ratio (OR), multivariable logistic regression odds ratio (MLR-OR) and their 95% confidence intervals (95% CI).

Exposure variables: having had at least one HIV indicator disease before HIV diagnosis (model 1); HIV testing after an HIV indicator disease (model 2) and type of indicator disease (grouped as: Hepatitis; Sexually Transmitted Infections (STI); Seborrhoeic dermatitis/Tuberculosis) (model 3).

Outcome variables: “Late presentation” was defined when the individual had a CD4 count <350 cells/mmc or an AIDS-defining event within 3 months of HIV diagnosis [17].

“Presentation with advanced HIV disease” was defined when the individual had a CD4 count <200 cells/mmc or an AIDS-defining event within 3 months of HIV diagnosis [17].

The covariates introduced in the multivariable models were: gender, age (as a continuous variable), area of birth (Italy versus another country), HIV exposure category (heterosexual contacts as a reference category) and a previous HIV- negative test.

In order to estimate the chance that a person with at least one HIV indicator disease will be diagnosed late, we calculated the positive predictive value (PPV) of the indicator diseases for late presentation.

Moreover, for 120 persons reporting more than one indicator disease, we performed the chi-square test to study the association between different combinations of multiple indicator diseases and late presentation.

We performed a multivariable regression analysis in order to compare the characteristics of individuals who were tested for HIV with those who were not, after the diagnosis of an indicator disease, adjusted for age, gender, CD4 cell count, area of birth, risk factors, previous negative test and type of indicator disease. A patient was classified as tested for HIV after an indicator disease if the patient first tested HIV-positive or tested negative within 6 months after the indicator disease. For individuals with more than one indicator disease, we considered the most recent one.

Since tuberculosis is considered an AIDS-defining illness, all statistical analysis were also performed excluding all individuals who reported tuberculosis in their clinical history.

According to data derived from the CASCADE collaboration on HIV seroconverters, we estimated the presumed time since HIV infection for each individual. For the purpose of the analysis we assumed that HIV infection occurred 1.19, 4.19 and 7.94 years before the first HIV positive test, respectively, for people with a CD4 cell count <500 cells/mmc, <350 cells/mmc and <200 cells/mmc at the time of HIV diagnosis [18]. Taking into account the estimated time since HIV infection, we analyzed data on individuals who reported the diagnosis of an indicator disease and were not tested for HIV to evaluate the proportion of individuals with late diagnosis who could have been diagnosed earlier if tested at the time the indicator disease was diagnosed.

Data were entered into an Access database, verified by double entry and analyzed using SPSS package (version 17.00 SPSS Inc., Chicago, Illinois).

## Results

### Occurrence of HIV indicator diseases

Out of the 1,864 individuals enrolled up to April 30, 2009, 1,735 had a CD4 cell count performed within 3 months of the first HIV- positive test and were included in the analysis. Late presenters were 51.3% (890/1735) and presenters with advanced HIV disease were 34.2% (594/1735). Table 1 shows the socio-demographic, clinical and epidemiological characteristics of the whole study population and late presenters. Five hundred individuals were foreign born (36% in Africa, 30% in South America; 20% in Eastern Europe) and had a median time of residence in Italy of 4 years (IQR: 1–9 years).

**Table 1:** Characteristics of study population and late presenters (CD4 <350 cells/mmc)

**Variable**	**Study population**		**HIV Late presenters**	
	**N = 1735**		**(CD4 <350 cells/mmc)**	
	**N (%)**		**N = 890**	
			**N (%)**	
**Median age** (min-max)	36 yy (18–86)	39 yy (18–86)		
**Gender**				
Male	1360	78.4%	676	76.0%
**Area of birth**				
Not Italy	500	28.8%	273	30.7%
**HIV exposure category**				
Heterosexual	695	40.1%	428	48.0%
MSM	835	48.2%	337	37.9%
IDU	136	7.8%	70	7.9%
Other/unreported	69	3.9%	55	6.2%
**Previous HIV**- **negative test**	780	45%	240	27.0%
**Previous HIV**- **negative test within 6 months**	111	6.4%	-	-
**CD4 cells**/**mmc**				
≥350	845	48.7%	-	-
200-349	296	17.1%	-	-
≤199 and AIDS	594	34.2%	-	-
**AIDS**	330	17.5%	324	36.4%
**HIV indicator disease**	504	29%	212	23.8%

Twenty-nine percent (504/1735) of the individuals reported at least one HIV indicator disease before HIV diagnosis and 120 reported more than one, for a total of 641 indicator diseases. Median time from indicator disease to HIV diagnosis was 15.4 months (IQR: 0.9-63.1 months).

Distribution of reported indicator diseases was: 210 viral hepatitis cases (126 HBV; 84 HCV); 382 STI cases (195 syphilis, 67 gonorrhoea, 33 genital herpes, 85 genital warts, 2 infectious vaginitis), 38 cases of seborrhoeic dermatitis and 11 cases of tuberculosis.

Concerning the 120 persons reporting more than one indicator diseases, 37 reported two or more STI, 49 reported STI and hepatitis, 20 reported both HBV and HCV infection and 14 reported seborrhoeic dermatitis/tuberculosis with a STI or one type of hepatitis.

In a subset of 683 individuals who completed a self-administered questionnaire, for each single indicator disease considered we observed a good concordance (k values of 0.7 to 0.9) between the questionnaire and the data collected at enrolment.

Among the 126 individuals who reported HBV infection, serological data were available for 113 and 95% of them (107/113) were HBsAg and/or HBcAb -positive. Among the 84 individuals who reported HCV infection, serological data were available for 79, and 90% of them (71/79) were anti-HCV- positive or HCV-RNA- positive.

**Figure 1:**
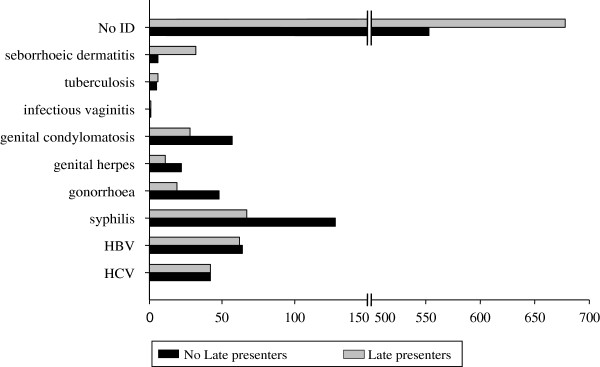
Distribution of indicator diseases by late presentation (CD4 < 350 cells/mmc or an AIDS-defining event).

Figure 1 shows the distribution of indicator diseases by late presentation (CD4 <350 cells/mmc or an AIDS-defining event).

### Association of indicator diseases with late presentation of HIV

Univariable regression analysis showed that individuals who reported at least one indicator disease before HIV diagnosis had a lower risk of late presentation (OR = 0.6; 95%CI: 0.5-0.7). The risk of late presentation significantly increased for older individuals (OR = 1.05 for each year of age; 95%CI: 1.04-1.06) and female gender (OR = 1.3; 95%CI: 1.1-1.7), while it decreased for MSM (OR = 0.4; 95%CI: 0.3-0.5), IDUs (OR = 0.7; 95%CI: 0.4-0.9) and individuals with a previous HIV negative test (OR = 0.2; 95%CI: 0.2-0.3). The above estimated associations were unchanged after all individuals who reported tuberculosis were excluded from the analysis.

With regard to the type of indicator disease, compared to individuals who did not report an indicator disease before HIV diagnosis, the risk of late presentation was lower for those reporting a STI (OR = 0.4; 95%CI: 0.3-0.5) while it increased for those reporting seborrhoeic dermatitis/tuberculosis (OR = 4.8; 95%CI: 2.0-11.4). Excluding all individuals with previous tuberculosis, the risk of late presentation for individuals reporting a STI was not modified, while it increased for those reporting seborrhoeic dermatitis (OR = 6.1; 95%CI: 2.1-17.5).

Multivariable logistic regression models, shown in Table 2, confirmed that the occurrence of at least one indicator disease was associated with a reduced risk of late presentation (MLOR = 0.7; 95%CI: 0.5-0.8) (model 1). Moreover, the probability of late presentation was significantly lower for individuals tested for HIV after an indicator disease (MLOR = 0.5; 95%CI: 0.4-0.7) compared with individuals who did not report a previous indicator disease (model 2).

**Table 2:** Multivariable regression models to estimate association of HIV indicator disease and late presentation

**Variable**	**Late presenters/Total (%)**	**MLOR (95%CI)**		
		**Model 1**^**a**^	**Model 2**^**b**^	**Model 3**^**c**^
**Age** (**per year old**)		**1**.**05** (**1**.**04**-**1**.**07**)	**1**.**05** (**1**.**04**-**1**.**07**)	**1**.**05** (**1**.**04**-**1**.**06**)
**Gender**				
Male	676/1360 (49.7%)	1	1	1
Female	214/375 (57%)	0.9 (0.7-1.2)	0.9 (0.6-1.2)	0.9 (0.7-1.2)
**Area of birth**				
Italy	617/1235 (50%)	1	1	1
Not Italy	273/500 (54.6%)	**1**.**5** (**1**.**2**-**1**.**9**)	**1**.**5** (**1**.**2**-**1**.**9**)	**1**.**5** (**1**.**2**-**1**.**9**)
**HIV exposure category**				
Heterosexual	428/695 (61.6%)	1	1	1
MSM	337/835 (40.3)	**0**.**5** (**0**.**4**-**0**.**7**)	**0**.**5** (**0**.**4**-**0**.**7**)	**0**.**6** (**0**.**4**-**0**.**7**)
IDU	70/136 (51.5%)	0.8 (0.5-1.2)	0.8 (0.5-1.2)	0.8 (0.5-1.1)
Other/unreported	55/69 (79.7%)	1.2 (0.7-2.1)	1.1 (0.6-2.1)	1.2 (0.7-2.1)
**Indicator Disease** (**ID**)				
No	678/1231 (55.1%)	1	-	-
Yes	212/504 (42.1%)	**0**.**7** (**0**.**5**-**0**.**8**)	-	-
**ID**^§^**and HIV test**				
No ID	678/1231(55%)	-	1	-
ID tested for HIV	129/358 (36%)	-	**0**.**5** (**0**.**4**-**0**.**7**)	-
ID not tested for HIV	71/123 (57.7%)	-	1.2 (0.8-1.8)	-
Missing data	12/23 (52.2%)	-	0.8 (0.3-1.9)	-
**Type of ID**^§^				
No ID	678/1231(55%)	-	-	1
STI*	95/302 (31.5%)	-	-	**0**.**5** (**0**.**4**-**0**.**6**)
Hepatitis	82/161 (50.3%)	-	-	0.8 (0.6-1.2)
Seb. dermatitis or TB**	35/41 (85%)	-	-	**4**.**3** (**1**.**7**-**10**.**6**)

statistically significant MLOR are shown in bold.

§ ID Indicator Diseases.

* STI: sexually transmitted infections.

** TB: Tuberculosis.

a: association of HIV indicator disease and late presentation adjusted for age, gender, area of birth, HIV exposure category and indicator disease.

b: association of HIV indicator disease and late presentation adjusted for age, gender, area of birth, HIV exposure category and HIV testing after an HIV indicator disease.

c: association of HIV indicator disease and late presentation adjusted for age, gender, area of birth, HIV exposure category and type of indicator disease.

If we consider the type of indicator disease, the multivariable analysis (model 3) showed that the risk of late presentation was significantly lower for individuals who reported diagnosis of a STI (MLOR = 0.5; 95%CI: 0.4-0.6) compared to individuals who did not report a previous indicator disease, while individuals with seborrhoeic dermatitis or tuberculosis had a higher risk of late presentation (MLOR = 4.2; 95%CI: 1.7-10.6). Excluding all individuals with tuberculosis from the analysis, the measures of association (MLOR) were unchanged in all 3 models, except for seborrhoeic dermatitis in model 3 (MLOR = 5.8; 95%CI: 2.0-17.0).

The multivariable regression analysis for presentation with advanced HIV disease confirmed the results of three multivariable regression models for late presenters (data not shown).

The overall PPV of these indicator diseases for late presentation was 0.4, varying from 0.9 for seborrhoeic dermatitis to 0.5 for hepatitis and 0.4 for STI. In particular, both for hepatitis and STI, women had higher chance to be HIV late presenter (PPV = 0.7, PPV = 0.5 respectively) than heterosexual men (PPV = 0.6, PPV = 0.4 respectively) and MSM (PPV = 0.4, PPV = 0.3 respectively).

Concerning the 120 persons reporting more than one indicator diseases, the proportion of late presenters who reported two or more STI (27.0%) was significantly lower than the proportion of those who reported STI and hepatitis (38.8%) or both HBV and HCV infection (65.0%), or seborrhoeic dermatitis/tuberculosis with a STI or one type of hepatitis (64.3%) (p = 0.013).

### Missed opportunities for timely HIV diagnosis following an indicator disease

Among the 504 individuals who reported at least one indicator disease, with respect to this diagnosis, 202 (40.1%) first tested HIV-positive within 6 months, 156 (30.9%) tested HIV- negative at least once in the following period and 123 (24.4%) were not tested for HIV following the indicator disease. For 23 (4.6%) individuals, information on previous HIV- negative tests was not available.

The multivariable regression analysis (Table 3) showed that individuals without a defined HIV exposure risk factor (classified as “other/unreported” mode of acquisition) were significantly less likely to be tested after an indicator disease than heterosexuals (MLOR = 0.15; CI 95%: 0.03- 0.84). On the contrary, those who had performed a previous HIV negative test were significantly more likely to be tested (MLOR = 3.9; CI 95%: 2.4 – 6.6). The regression analysis results were not affected by the exclusion of patients with tuberculosis.

**Table 3:** **Multivariable regression model to compare characteristics of individuals tested for HIV after an HIV indicator disease vs**. **individuals not tested after an HIV indicator disease**

**Variable**	Tested for HIV after indicator disease/Total N = 481 (%) *	**MLOR (95%CI)**
**Median age** (**min**-**max**)	36 (20–86)	0.99 (0.97-1.01)
**Median CD4** (**min**-**max**)	416 (2–1458)	1.001 (1–1.002)
**Gender**		
Female	35/51 (68.6%)	1
Male	323/430 (75.1%)	0.7 (0.3-1.6)
**Area of birth**		
Italy	278/379 (73.4%)	1
Not Italy	80/102 (78.4%)	1.7 (0.9-3.1)
**HIV exposure category**		
Heterosexual	80/113 (70.8%)	1
MSM	239/311 (76.8%)	0.7 (0.3-1.3)
IDU	35/47(74.5%)	0.5 (0.2-1.2)
Other/unreported	4/10 (40%)	**0**.**2** (**0**.**03**-**0**.**8**)
**Previous negative test**		
No	93/163 (57.1%)	1
Yes	248/297 (83.5%)	**4**.**1** (**2**.**5**-**6**.**8**)
**Type of indicator diseases**		
STI	220/298(73.8%)	1
Hepatitis	113/143 (79.0%)	1.7 (0.9-3.1)
Seborrhoeic dermatitis or TB**	25/40 (62.5%)	1.3 (0.6-3.2)

statistically significant MLOR are shown in bold.

* The 23/504 subjects with an HIV indicator disease were excluded from analysis because not all data were available.

** TB: Tuberculosis.

Based on our assumption of the presumed time since HIV infection, 59 individuals (3.4% of the study population) who were diagnosed with an indicator disease and were not tested for HIV thereafter could already have been HIV- infected at the time of the indicator disease and their diagnosis could have been anticipated a median of 18.6 months.

Among the 890 late presenters (CD4 < 350 cells/mmc), 52 individuals (5.8%) could already have been infected with HIV at the time of the indicator disease, which was diagnosed a median of 22.6 months before HIV. The majority of these 52 individuals were male (84.6%) with a median age of 39 years (range 22–71 years) and Italian (88.5%) With regard to HIV exposure category, individuals infected through homosexual contacts accounted for 57.7%, heterosexual contacts accounted for 28.8%, and those through injecting drug use for 9.6% of the total. More than one half (31/52) reported a STI as an indicator disease and 44.2% were never tested before HIV diagnosis. An additional 27 (3%) late presenters reported an indicator disease before HIV diagnosis that, according to our assumption, occurred before the estimated time of HIV infection.

Among the 594 presenters with advanced HIV disease (CD4 < 200 cells/mmc), 33 (5.5%) could already have been infected at the time of the indicator disease which was diagnosed a median of 41.6 months before HIV.

## Discussion

Routine HIV testing in individuals presenting with diseases/conditions which may indicate the presence of HIV infection, the so-called indicator diseases, has been recommended to reduce undiagnosed HIV infection [11,12]. In particular, according to the guidance outlined by the HIV in Europe initiative, routine testing of any person presenting with a condition associated with an undiagnosed HIV prevalence of >0.1% is cost-effective and promotes earlier diagnosis of HIV infection [12].

In our multi-centre study, we found that almost thirty percent of newly diagnosed adults with HIV infection reported at least one indicator disease before HIV diagnosis, and that being tested for HIV within six months of being diagnosed with an indicator disease reduced the risk of late HIV presentation by 50%.

More than half of our study population had a late HIV diagnosis, i.e. at a stage of their disease when, according to current guidelines, they should have already started cART, and 39% of the individuals were first diagnosed with HIV at an advanced stage. These figures are consistent with previous reports from industrialized countries. In Europe among 10,222 newly diagnosed HIV infection cases with CD4 cell counts reported in 2009, 51% had a first CD4 cell count below 350/mmc [4] and a series of surveys show that 29–39% of individuals with a new HIV diagnosis have less than 200 CD4 cells/mmc at first presentation [19]. Similarly, more than half of the individuals enrolled in cohort studies in North America from 1997–2007 had fewer than 350 CD4 cells/mmc when they first presented for HIV care [6].

Factors associated with late diagnosis and presentation with advanced HIV disease in our study included older age, being foreign born and having acquired HIV infection through heterosexual contact. These results agree with those from previous studies [20-22] and suggest that individuals perceiving themselves as being at risk of infection, such as MSM and/or IDUs, are more likely to be diagnosed earlier than individuals who are not tested until the clinician evaluates the situation and recommends testing, such as foreigners and older people.

On the other hand, we found that a previous diagnosis of an HIV indicator disease followed by an HIV test within six months significantly reduced the risk of late presentation.

Almost thirty percent of our study population reported at least one indicator disease before HIV diagnosis and more than ten percent were diagnosed with HIV within six months of being diagnosed with an indicator disease. Taken together, these data support the hypothesis that testing for HIV following a diagnosis of an indicator disease may significantly prevent late diagnosis.

To our knowledge, few reports have analyzed the association between diagnosis of an indicator disease and probability of receiving an earlier HIV diagnosis [23-28]. Klein et al. have reviewed medical encounters before HIV diagnosis in the US concluding that increased recognition of clinical indicators for HIV testing prompted earlier HIV diagnosis in 22% of individuals [23]. Recently, Ellis S. et al. found that in the United Kingdom, among 1,112 newly diagnosed HIV-infected patients, a quarter of them were identified as having missed an opportunity for earlier diagnosis [24]. Similar to our results, Lo YC et al. found that in Taiwan, missed opportunities for HIV testing were more common in individuals with late diagnosis than in those with earlier diagnosis (23% vs. 15.8%), and individuals with late HIV diagnosis were more likely than their counterparts to have received a diagnosis of seborrhoeic dermatitis (7.4% vs. 0.8%, p = 0.02) for which HIV testing was not offered by the health care provider [25].

In contrast with these results, in the study conducted between 2001 and 2005 in a South Carolina health care facility, Duffus et al. found that approximately 80% of the health care visits before HIV diagnosis for both late and early testers were for conditions not likely to prompt HIV testing in a non-routine testing environment [26]. The authors concluded that a clinical risk-based testing strategy, even if implemented successfully in their facility, would still have missed an earlier diagnosis the majority of the time.

In our study, the association with late diagnosis differed according to the type of indicator disease considered. Individuals reporting a STI had the lowest risk of late diagnosis, while those reporting tuberculosis and seborrhoeic dermatitis had an increased chance of being diagnosed at an advanced stage of immunosuppression. This finding is not surprising since the risk of persons with HIV developing tuberculosis significantly increases, parallel to their increasing level of immunodeficiency [29]. Nevertheless, it should be emphasized that patients with tuberculosis should be routinely tested for HIV, since failure to diagnose and treat HIV in these patients could be detrimental to survival [30]. It has also been shown that seborrhoeic dermatitis generally occurs in individuals with CD4 cell counts between 201–500 cells/mmc [31].

In our study population, although diagnosis of an indicator disease reduced the overall risk of late diagnosis, almost one fourth of the individuals were not tested for HIV within six months of being diagnosed with the indicator disease.

The probability of being tested for HIV following an indicator disease didn’t differ for age, gender, CD4 cell count or place of birth. Individuals who reported to have been previously tested for HIV had a higher probability of being tested after an indicator disease; on the contrary, those with an unreported mode of HIV acquisition had a lower probability of being tested after an indicator disease.

It is difficult to interpret this result since individuals with unreported modes of HIV acquisition may actually include people with different risk behaviours [32]. However, one may speculate that individuals classified in this group may be less likely to disclose risk behaviours to the health care provider, thus not perceived as being at risk and consequently less likely to be offered HIV testing. Testing practices are highly dependent on the local culture of the clinics, the individual practices of health care workers and on perception of risk at an individual level, often resulting in a missed opportunity to prevent late diagnosis. Among US adults with positive serologic test results for HBV and/or HCV, Krain et al. found that only 40% had been tested for HIV and that older individuals were less likely to be tested [33]. It has been shown in a number of studies that people presenting with severe HIV-related diseases frequently had a history of repeated previous contacts with medical services, both in primary and secondary care, but were not tested for HIV [7,23,28]. This may reflect, at least in some instances, attitudinal barriers of health care provider to offer test rather than low patient acceptability [9;10]. In an indicator condition guided HIV testing strategy, all patients presenting to any health care setting with specific indicator conditions would be offered HIV testing independent of risk assessment, as part of routine care.

Regarding the potential further impact of this strategy, our data suggest that HIV diagnosis could have been anticipated by a median of 22.6 months in almost 6% of late presenters if they had been tested at the time the indicator disease was diagnosed. We may have underestimated this proportion. In fact, an additional 3% of late presenters reported an indicator disease which occurred before our estimated time of HIV infection, based on CD4 cell counts at HIV diagnosis. Since there is variability in progression of HIV infection, not taken into account in our analysis, it is possible that some of these individuals could already have been infected at the time the indicator disease was diagnosed.

The indicators diseases considered in our study, are conditions that a population-based case control study conducted in Denmark [34] and the HIDES study (HIV Indicator Diseases Across Europe Study) [9], showed to be associated with HIV prevalence greater than 0.1%. The above studies, that were published when our analysis was already completed, have shown that an HIV prevalence above this threshold may be observed in a series of clinical conditions that were not included in our analysis, probably resulting in a underestimation of the overall impact of this policy in preventing late HIV diagnosis.

Other potential limitations of our study need to be considered. First, HIV-testing history and clinical history were collected by patient interviews and this may imply a recall bias. However, we found a good concordance between the information on indicator diseases collected at enrolment and those reported in a self-administered questionnaire. Additionally, serological data provided evidence of past HBV or HCV infection, respectively in 95% and 90% of individuals who reported these infections at enrolment. Second, for individuals who were not tested for HIV following an indicator disease, we do not know if HIV testing was recommended by the health care provider but was refused by the patient. Third, our study population accounts for approximately 60% of the newly diagnosed HIV infections reported every year to the Regional HIV Surveillance System in Lazio region [13] and therefore may not be fully representative.

## Conclusions

A recent survey conducted in European countries has shown that an HIV prevalence greater than 0.1% can be recorded among patients presenting with some indicator medical conditions, including those considered in the present study [9]. Based on these results, the HIV in Europe initiative strongly recommended routine HIV testing of individuals presenting with these conditions, independent of any risk assessment which may be a cost-effective intervention to identify persons living with undiagnosed HIV infection [12].

Our data suggest that testing for HIV following diagnosis of an indicator condition significantly decreases the probability of late HIV diagnosis, and thus reinforces the indication to implement this strategy as an important component of a control policy for the HIV epidemic.

However, this policy needs to be complemented by client-centred interventions that aim to increase awareness in people who do not perceive themselves as being at risk for HIV.

## Abbreviations

cART: Combination antiretroviral therapy; US: United States; CDC: Centres for Disease Control and Prevention; IDU: Intravenous drug use; MSM: Men who have sex with men; HBV: Hepatitis B virus; HCV: Hepatitis C virus; STI: Sexually Transmitted Infections; MLR-OR: Multivariable logistic regression odds ratio; PPV: Positive predictive value.

## Competing interests

EG has received honoraria for presentations at workshops and/or travel grants from Abbott, Bristol-Myers Squibb, Boheringer Ingelheim. PS, GC, GDC, MG, CMM, SAB, ARB, SG, AS, AC, NO, VP and GI declare that they have no competing interest.

## Authors’ contributions

EG designed the study and drafted the manuscript. PS participated in the design of the study and drafted the manuscript. GC conducted the statistical analysis and contributed to writing the manuscript. GDC; MG; CMM; SAB; ARB; SG; AS; AC; NO contributed with data collection and critically revised the manuscript. VP; GI participated in the design of the study and critically revised the manuscript. All authors have read and approved the final manuscript.

## Pre-publication history

The pre-publication history for this paper can be accessed here:

http://www.biomedcentral.com/1471-2334/13/473/prepub

## Supplementary Material

Additional file 1 SENDIH Study questionnaire. Click here for file

## The SENDIH (Studio sull’Epidemiologia delle Nuove Diagnosi di Infezione da HIV) Study Group

Co-ordinating Centre

E. Girardi, N. Orchi, C. Angeletti, R. Balzano, P. Elia, A. Navarra, G. Nurra, A. Palummieri, (‘L. Spallanzani’ National Institute for Infectious Diseases, IRCCS, Rome)

Participating Physicians and Centres

L. Alba, A. Ammassari, A. Antinori, F. Baldini, R. Bellagamba, N. Bevilacqua, E. Boumis, M.R. Capobianchi, S. Cerilli, P. Chinello, A. Corpolongo, R. D’Arrigo, G. De Carli, G. D’Offizi, F. Forbici, F.M. Fusco, V. Galati, P. Ghirga, L. Giancola, C. Gori, S. Grisetti, F.N. Lauria, G. Liuzzi, P. Marconi, A. Mariano, P. Narciso, E. Nicastri, P. Noto, A. F. Palmieri, C.F. Perno, N. Petrosillo, R. Pisapia, S. Pittalis, V. Puro, A. Sampaolesi, P. Scognamiglio, M.R. Sciarrone, M. Selleri, C. Sias, S. Topino, V. Tozzi, L. Vincenzi, U. Visco Comandini, C. Vlassi, M. Zaccarelli, S. Zaniratti

(“L. Spallanzani” National Institute for Infectious Diseases, IRCCS, Rome, Italy), V. Vullo, M. Falciano (CRAIDS Policlinico Umberto I, Rome, Italy), M. Andreoni, L. Sarmati, A.R. Buonomini (Clinical Infectious Diseases Unit, Tor Vergata University, Rome, Italy), A. Di Carlo, M. Giuliani (San Gallicano Dermatological Institute (IRCCS), Rome, Italy), R. Brancatella, T. Maggi, F. Errico (U.O. AIDS ASL RMB, Rome, Italy), A. De Filippis, R. Di Bacco (U.O. AIDS ASL RMC, Rome, Italy), S. Schito (U.O. AIDS ASL RMD, Ostia, Italy), P. Gattari, L. Spizzichino (U.O. AIDS ASL RME, Rome, Italy), M. Francesconi, G. Pace (U.O. AIDS ASL RMH, Frascati, Albano, Italy), I. Gallo, E. Anzalone (CRAIDS Hospital, Frosinone, Italy), C.M. Mastroianni, L. Tacconi, V.S. Mercurio, M. Lichtner (CRAIDS Hospital S. Maria Goretti, Latina, Italy), G. Natalini Raponi, A. Pitorri (CRAIDS S. Camillo de Lellis Hospital, Rieti, Italy), A. Caterini, S. Aviani Barbacci, (CRAIDS Bel Colle Hospital, Viterbo, Italy).
