# Supplementary material for: The potential impact of routine testing of individuals with HIV indicator diseases in order to prevent late HIV diagnosis
Source: BMC Infect Dis. 2013 Oct 10;13:473. doi: 10.1186/1471-2334-13-473 (PMC3852490; doi:10.1186/1471-2334-13-473)
Supplement: Additional file 1 — SENDIH Study questionnaire. [file 1471-2334-13-473-S1.doc]

***
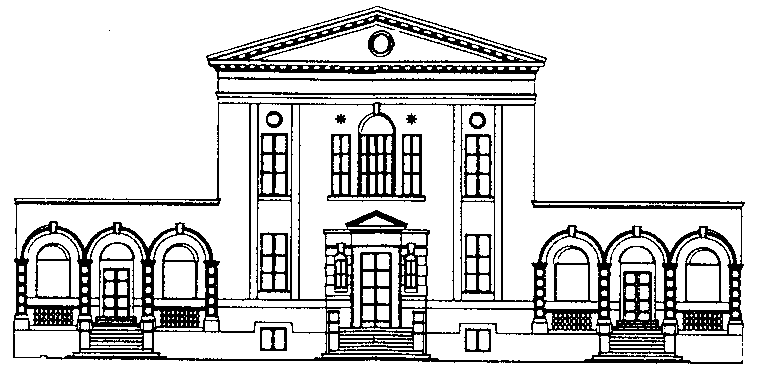
***

**Istituto Nazionale per le Malattie Infettive**

**"Lazzaro Spallanzani"**

Istituto di Ricovero e cura a Carattere Scientifico

A MULTICENTRIC TRANSVERSAL STUDY

OF THE EPIDEMIOLOGICAL AND BEHAVIORAL CHARACTERISTICS OF PERSONS NEWLY DIAGNOSED WITH HIV INFECTION

QUESTIONNAIRE

v. 2.2

Part A

This questionnaire is divided into five sections.

Parte A contains personal information and must be filled out and completed by the referring physician for each newly diagnosed infection, even if the patient refuses to participate in the study or is lost at follow-up.

Therefore, every newly diagnosed patient must be assigned a seven-digit identification code (diagnostic centre monitoring card).

Part A, properly filled out, even if the subsequent sections are not filled out, can be sent to the coordinating centre - INMI L. Spallanzani, Dipartimento Epidemiologico e di Ricerca Pre-clinica (INMI L. Spallanzani, Department of Epidemiology and Pre-clinical Research)- even by fax, to the following number:06 5582825.

At the end of Part A, if there are no hindrances of any kind in administering this questionnaire and if the patient accepts, you may proceed to the other sections (Parts B, C, D, E). Please use the same identification code in part A on the rest of the questionnaire.

PART A – PERSONAL INFORMATION

**A1. Patient Code** |__|__|__|__|__|__|__| **A2. Date administered** |__|__| |__|__| |__|__|__|__|

*day month year*

**A3. Centre**__________________________________________________________________________________

###### A4.Name of Interviewer _______________________________________________________________________

**A5. Gender** 🞎 **F** 🞎 **M** **A6.** **Year of Birth** |__|__|__|__|

**A7. Country of Birth** _________________________ **A8. If born abroad, has been in Italy for** |__|__| **years**

**A9. If you are an immigrant, do you have a legal permit of stay**? 🞎**YES** 🞎 **NO**

**A10. Province of Birth** ________________________ **A11. Province of Address**_________________

**A12. Civil Status**

🞎 Single 🞎 Married 🞎 Separated

🞎 Living together 🞎 Divorced 🞎 Widow

**A13. Date of 1^st^ HIV** **positivity** |__|__| |__|__| |__|__|__|__|

*day month year*

###### A14. Were previous HIV tests negative? 🞎YES 🞎 NO

**A15. If yes, last date tested negative** |__|__| |__|__| |__|__|__|__| 🞎 Referred

*day month year*  🞎 Documented

**A16. Seroconversion taking place**? **🞎 YES 🞎 NO If YES, Data WB indeterminata** |__|__| |__|__| |__|__|__|__|

or HIV Ag positive day month year

**A17. Acute infection**  **🞎 YES🞎** **NO** **If YES Date symptoms began**|__|__| |__|__| |__|__|__|__|

*day month year*

**A18. Absolute CD4 values at the time of 1st HIV positivity |__|__|__|__|/mmc**

**A19. Plasma Viremia at the time of 1st HIV positivity** |__|__|__|__|__|__|__|__| (copies/ml)

**A20. Before being diagnosed with HIV infection was the patient ever diagnosed with any of the following diseases? :** *(more than one response is possible)*

*month year* *month year*

🞎 Hepatitis C when?|__|__| |__|__|__|__| 🞎 Seborrheic Dermatitis when?|__|__| |__|__|__|__|

🞎 Hepatitis B when?|__|__| |__|__|__|__| 🞎 Tuberculosis when?|__|__| |__|__|__|__|

🞎 Syphilis when?|__|__| |__|__|__|__| 🞎 Genital Herpes when ?|__|__| |__|__|__|__|

🞎 Gonorrhea when?|__|__| |__|__|__|__| 🞎 Condyloma when?|__|__| |__|__|__|__|

🞎 Infectious Vaginitis when?|__|__| |__|__|__|__| 🞎 None of the above

**A21. Means of transmission (physician**’**s evaluation)**

🞎 Homo/bisexual Sex 🞎 Heterosexual Sex 🞎 Use of I.V. drugs

🞎 Transfusions/Blood Products 🞎 Undefined Risk

🞎 Other *(specify)*____________________________________

**A22. Positivity of Partner** 🞎**YES** 🞎 **NO** 🞎 **UNKNOWN**

**A23. Has AIDS been diagnosed**? 🞎**YES** 🞎 **NO**

**If yes,**

###### A24.Month/year of AIDS diagnosis |__|__| |__|__|__|__|

*month year*

A25. Pathologies indicative of AIDS ___________________________________________________________

___________________________________________________________

A26. Was it possible to administer the questionnaire? 🞎 YES 🞎 NO

A27. If No,why?:

🞎 subject refused

🞎 subject lost at observation

🞎 subject deceased

🞎 subject already discharged from the ward

🞎 subject with severe neurological pathologies

🞎 subject doesn’t understand Italian

🞎 Other (specify)

A28. Was it possible to take a blood sample? 🞎 YES 🞎 NO

A29.If No, why?: A.28.If yes, date of blood sample |__|__| |__|__| |__|__|__|__|

🞎 subject refused day month year

🞎 subject lost at observation

🞎 subject deceased

🞎 subject already discharged from the ward

🞎 Other (specify)

***
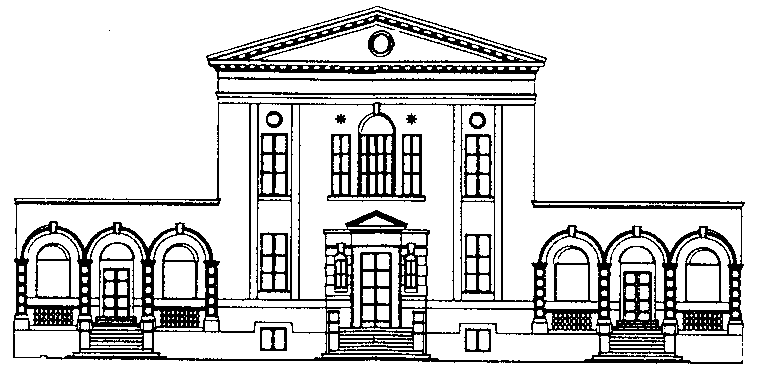
***

**Istituto Nazionale per le Malattie Infettive**

**"Lazzaro Spallanzani"**

Istituto di Ricovero e cura a Carattere Scientifico

A MULTICENTRIC TRANSVERSAL STUDY

OF THE EPIDEMIOLOGICAL AND BEHAVIORAL CHARACTERISTICS OF PERSONS NEWLY DIAGNOSED WITH HIV INFECTION

QUESTIONNAIRE

v.2.2

The data from epidemiological surveys do not allow for defining the most recent dynamics of the HIV epidemic. Some studies show that there are heterogeneous risk behaviors even within groups of populations that have acquired HIV infection in the same way. The aim of this study is to outline a behavioral profile of subjects with new or recently diagnosed infections.

How to fill out the questionnaire

The questionnaire must be filled out in all five parts with the aid of an interviewer.

Part A contains personal data, and must be filled out by the referred physician. At the end of Part A, if there are no hindrances of any kind in administering this questionnaire, and if the patient accepts, you may proceed to the other sections (Parts B, C, D, E).

It is desirable that the questionnaire be administered at the visit following communication of HIV-positive status or upon hospital discharge, for hospitalized patients.

The questionnaire will be identified by a code, not a name.

**PATIENT CODE** |__|__|__|__|__|__|__|  **DATE ADMINISTERED** |__|__| |__|__| |__|__|__|__|

*day month year*

Centre _____________________________________________________________________________

PART B –SOCIO-DEMOGRAPHIC INFORMATION

**B1. Years of Education**

🞎 0 years 🞎 1-5 years 🞎 6-8 years

🞎 9-13 years 🞎 >14 years

**B2. Professional Status**

🞎 Employed 🞎 Unemployed

🞎 Student 🞎 Housewife

🞎 Retired 🞎 Other_________________________________

###### B3. In the last 6 months, what was your primary source of income?

🞎 Employee 🞎 Odd Jobs

🞎 Self-Employed 🞎 Retired –Invalid

- Other (*specify*) ____________________

**B4. In the last 6 months, your monthly wage was equal to:**

🞎 0 🞎 less than 500 Euros

🞎 between 500 and 1000 Euros 🞎 between 1000 and 2000 Euros

🞎 more than 2000 Euros 🞎 no response

###### B5. In the last 6 months, where did you reside most of the time?

🞎 Own home 🞎 With relatives or friends

🞎 Guest House 🞎 Guest in a community

🞎 In Prison 🞎 Homeless

🞎 Other (*specify*) _____________________

###### B6. In the last 6 months, who did you live with most of the time?

🞎 Alone 🞎 With relatives (parents,siblings, cousins, aunts/uncles….)

🞎 With my partner 🞎 With people other than relatives (shared living…)

🞎 With friends 🞎 With my children

🞎 Other____________________________________

**B7.Do you have children?** 🞎 YES 🞎 NO

**If YES** how many **|__|**

**Age |___| Does he/she live with you?** 🞎YES 🞎 NO

**Age |___| Does he/she live with you?** 🞎YES 🞎 NO

**Age |___| Does he/she live with you?** 🞎YES 🞎 NO

**Age |___| Does he/she live with you?** 🞎 YES 🞎 NO

**PART C –TEST AND INFECTION HISTORY**

**C1. Did you ever take an HIV test that resulted negative?** 🞎 YES 🞎 NO

**C2.If YES, how many?**

🞎 1 🞎 2-3

🞎 4-5 🞎 more than 5

C3.If YES, in what month and year did you take the last HIV test that resulted negative?

|__|__| |__|__|__|__|

*month year*

**If at least 12 months have passed since the last negative test:**

**C4. Why didn’t you take other tests?**

**🞎 I didn’t have other risky behavior**

**🞎 The partners I had seemed healthy**

**🞎 I thought that if I had HIV-positive partners they would have told me**

**🞎 I have/had a stable sexual relationship**

**C5. If NO, why didn’t you ever take the test before?**

**🞎 I didn’t know that I was at risk**

**🞎 Fear of the result**

**🞎 Knowing that I was HIV-positive wouldn’t have improved my situation**

**🞎 Other *(specify)* _____________________**

**C6. Why did you take the test that resulted positive?**

🞎 Because I wasn’t feeling well 🞎 Pregnancy

🞎 For surgery 🞎 To find out if I was HIV-positive

🞎 At -risk behavior 🞎 Because it was prescribed to me

🞎 Other *(specify)*__________________________________________________

**C7.Who suggested you take it?**

🞎 Family Physician 🞎 Medical Specialist

🞎 AIDS Hotline 🞎 Friend

🞎 Partner 🞎 Relative

🞎 Surgeon before an operation 🞎 Gynecologist for pregnancy

🞎 No one (I thought of it myself) 🞎 Volunteer organization worker

🞎Other (*specify* ) __________________________________

C8. Where did you take it?

🞎 Public Out-patient Clinic / Infectious Diseases 🞎 While hospitalized

🞎 Centre for sexually transmitted diseases 🞎 Ser.T

- Private laboratory 🞎 In prison
- Other *(specify)_*____________________

C9. How did you receive the results?

- Communicated while speaking with the doctor and/or psychologist 🞎 In a closed envelope

🞎 Communicated by another healthcare worker 🞎 I don’t remember

🞎 Other*(specify)_*________________________

**C10. In what month/ year do you think you were infected?** |__|__| |__|__|__|__| 🞎 I don’t know

month year

**C11.How do you think you became infected?**

🞎 Heterosexual sex with a regular partner (> 3months) 🞎 Heterosexual sex with an occasional partner

🞎 Homosexual sex with a regular partner (> 3 months) 🞎 Homosexual sex with an occasional partner

🞎 Transfusions/Blood products 🞎 Use of syringes for drugs

🞎 Paid Sex 🞎 I don’t know

🞎 Other (*specify)*______________________________

###### C12. Where do you think you contracted the infection?

🞎 In Italy 🞎 Abroad *(specify the country) _______________* 🞎 I don’t know

**C13. If you contracted the infection through sexual intercourse, did or does the partner who transmitted the infection to you inject drugs?**

🞎 YES 🞎 NO 🞎 I don’t know

**C14. If you contracted the infection through sexual intercourse, did the partner who transmitted it to you know he/she was HIV-positive?**

🞎 YES 🞎 NO 🞎 I don’t know

**C15. If YES, did he/she tell you?**

🞎 YES 🞎 NO

**C16. Did the partner who transmitted the infection take or was he/she taking antiretrovirals?**

🞎 YES 🞎 NO 🞎 I don’t know

**C17. Before discovering that you were HIV-positive, did you ever have one or more of these problems?**

*(more than one response is possible)*

month year

🞎 Fever for a long period of time (more than one month) when? |__|__| |__|__|__|__|

🞎 Diarrhea for a long period of time (more than one month) when? |__|__| |__|__|__|__|

🞎 Weight loss (unintentional weight loss of > 10% in one month) when? |__|__| |__|__|__|__|

🞎 Swollen glands in various parts of the body when? |__|__ | |__|__|__|__|

🞎 Herpes Zoster (Shingles) when? |__|__| |__|__|__|__|

🞎 Pneumonia when? |__|__ | |__|__|__|__|

🞎 Sexually transmitted diseases when? |__|__| |__|__|__|__|

🞎 Skin disorders (blotches and/or reddening of the skin, itching) when? |__|__| |__|__|__|__|

🞎 Evident difficulty concentrating or remembering when? |__|__| |__|__|__|__|

🞎 None of the above

**C18. Did you ever see a doctor for these problems?**

🞎 YES 🞎 NO

C19.If YES, Who?

🞎 Family Physician 🞎 Infectivologist

🞎 Dermatologist 🞎 Other *Specialist*____________________________

**C20. Before knowing that you were HIV-positive were you ever diagnosed with one or more of the following diseases?** *(more than one response is possible)*

*month year* *month year*

🞎 Hepatitis C when?|__|__| |__|__|__|__| 🞎 Seborrhoic Dermatitis when?|__|__| |__|__|__|__|

🞎 Hepatitis B when?|__|__| |__|__|__|__| 🞎 Tuberculosis when?|__|__| |__|__|__|__|

🞎 Syphilis when?|__|__| |__|__|__|__| 🞎 Genital Herpes when?|__|__| |__|__|__|__|

🞎 Gonorrhea when?|__|__| |__|__|__|__| 🞎 Condyloma when?|__|__| |__|__|__|__|

🞎 Infectious Vaginitis when?|__|__| |__|__|__|__| 🞎 None of the above

###### C21. In the year before discovering you were HIV-positive, were you ever hospitalized?

###### 🞎 YES 🞎 NO 🞎 I don’ t remember

C22. If YES, in which ward? (i.e. General Medicine, Surgery, Infectious Diseases) _____________________

**C23. With what diagnosis**________________________________ **date admitted**|__|__| |__|__|__|__|

month year

###### C24. Do you usually cure yourself with natural medicines (Phytotherapy, Homeopathy, Acupuncture, etc.)? 🞎 YES 🞎 NO

**PART D – SEXUAL BEHAVIOR AND DRUG USE**

**D1.Have you ever had sexual intercourse?**

🞎YES 🞎 NO

**If your answer is** NO **go directly to query D.25**

**D2. How many sexual partners have you had in your life?**

🞎 none 🞎 one 🞎 from two-three

🞎 from four - five 🞎 from six-ten 🞎 from eleven - twenty-five

🞎 more than twenty-five

**D3. Did you ever have sex with someone of the same sex?**

🞎 YES 🞎 NO

**D4.If YES:**

🞎 once 🞎 rarely 🞎 regularly

**D5. Do you have a regular partner (one you consider your main partner and with whom you are involved in a relationship for at least three months)?**

🞎 YES 🞎 NO

## D6. Did your regular partner ever get tested for HIV? 🞎 YES 🞎 NO 🞎 I don’t know

## D7.If YES, what was the result? 🞎 Positive 🞎 Negative 🞎 I don’t know

**D8. At the beginning of your relationship, did you or your partner get tested for HIV?**

🞎 YES 🞎 NO

**D9a. If YES, who took the test?**

🞎 **Both**

🞎 **Only you**

🞎 **Only your partner**

**D9b. If NO, why didn’t you take the test?**

🞎 **We were both sure we were negative**

🞎 **We trusted each other**

🞎 **Fear of being judged by the other**

🞎 **Knowing the HIV status of my partner would not have affected our relationship**

## D10. Does your regular partner inject drugs? 🞎 YES 🞎 NO 🞎 I don’t know

**D11. If YES, does he/she use needles used by others?**

🞎 YES 🞎 NO 🞎 I don’t know

**D12. In the 12 months before discovering you were HIV-positive, how many sexual partners did you have?**

🞎 none 🞎 one 🞎 two-three

🞎 four-five 🞎 more than five

**If your answer was** none **go directly to query** **D.25**

**D13. In the 12 months before discovering you were HIV-positive, did you have sex with occasional partners who were drug addicts?**

🞎 YES 🞎 NO 🞎 I don’t know

**D14. In the 12 months before discovering you were HIV-positive did you have sex with your regular partner?**

🞎 YES 🞎 NO

**D15.If YES, did you use a condom when you:**

|  | ***I don’t* *remember*** | ***never*** | ***sometimes*** | ***always*** | ***I didn’t have this kind of sex*** |
| --- | --- | --- | --- | --- | --- |
| had vaginal sex | ⭘ | ⭘ | ⭘ | ⭘ | ⭘ |
| had anal sex | ⭘ | ⭘ | ⭘ | ⭘ | ⭘ |
| gave oral sex | ⭘ | ⭘ | ⭘ | ⭘ | ⭘ |
| received oral sex | ⭘ | ⭘ | ⭘ | ⭘ | ⭘ |

**D16. If you didn’t use a condom, why?**

- **I didn’t have one at the time**
- **It is expensive**
- **I don’t like to use them**
- **My partner doesn’t like them**
- **I used another contraceptive method (specify which)_____________________-**
- **I didn’t think it was risky**
- **I wasn’t asked/it wasn’t proposed**
- **I trusted that my partner was HIV-negative**
- **I find it hard to propose**
- **I expect my partner to ask**
- **Other (specify)________________________**

**D17. In the 12 months before discovering you were HIV-positive, did you have sex with occasional partners?**

🞎 YES 🞎 NO

**D18. If YES, did you use a condom when you:**

|  | ***I dont remember*** | ***never*** | ***Sometimes*** | ***always*** | ***I didnt have this kind of sex*** |
| --- | --- | --- | --- | --- | --- |
| had vaginal sex | ⭘ | ⭘ | ⭘ | ⭘ | ⭘ |
| had anal sex | ⭘ | ⭘ | ⭘ | ⭘ | ⭘ |
| gave oral sex | ⭘ | ⭘ | ⭘ | ⭘ | ⭘ |
| received oral sex | ⭘ | ⭘ | ⭘ | ⭘ | ⭘ |

**D19. If you didn’t use a condom, why?**

- **I didn’t have one at the time**
- **It is expensive**
- **I don’t like to use them**
- **My partner doesn’t like them**
- **I used another contraceptive method (specifywhich)_____________________-**
- **I didn’t think it was risky**
- **I wasn’t asked/it wasn’t proposed**
- **I trusted that my partner was HIV-negative**
- **I find it hard to propose**
- **I expect my partner to ask**
- **Other (specify)__________________________**

**D20. In the last 12 months, where did you most often meet your partner? (more than one response is possible)**

🞎 in private 🞎 sauna/gym 🞎 discoteque 🞎 public parks /street

🞎 in gay clubs 🞎 after internet contact 🞎 other (specify)____________________

**D21. In the 12 months before discovering you were HIV-positive, did you ever pay to have sex?**

🞎 YES 🞎 NO

**D22. In the 12 months before discovering that you were HIV-positive, were you ever paid to have sex?**

🞎 YES 🞎 NO

**D23.If YES, did you use a condom during these sexual encounters?**

🞎 never 🞎 sometimes 🞎 always 🞎 I don’t remember

**D24.If you didn’t use a condom, why?**

- **I didn’t have one at the time**
- **It is expensive**
- **I don’t like to use them**
- **My partner doesn’t like them**
- **I used another contraceptive method (specify which)_____________________-**
- **I didn’t think it was risky**
- **I wasn**’**t asked/it wasn**’**t proposed**
- **I trusted that my partner was HIV-negative**
- **I find it hard to propose**
- **I expect my partner to ask**
- **Other (specify)__________________________**

**D25. How would you define your sexual preferences?**

🞎 heterosexual 🞎 homosexual 🞎 bisexual

**D26. Before being diagnosed HIV-positive did you ever use drugs?** 🞎YES 🞎 NO

**D27.If YES, which ones?** *(more than one response is possible)*

🞎 Heroin 🞎 Cocaine 🞎 Cannabis 🞎 LSD 🞎 Ecstasy 🞎 Other*___________*____

**D28. Before being diagnosed HIV-positive did you ever inject drugs?**  🞎 YES 🞎 NO

**D29.If YES, did you use needles used by others?** 🞎YES 🞎 NO

**D30. Have you used drugs in the past 12 months?** 🞎YES 🞎 NO

**D31. If YES, which?** *(more than one response is possible)*

🞎 Heroin 🞎 Cocaine 🞎 Cannabis 🞎 LSD 🞎 Ecstasy 🞎 Other*___________*____

**D32. In the past 12 months did you inject drugs?** 🞎YES 🞎 NO

**D33.If YES, did you use needles used by others?** 🞎YES 🞎 NO

**D34. In the 12 months before being diagnosed HIV-positive, did you drink 3 or more glasses of alcoholic beverages per day?**

🞎 Never

🞎 More than 3 days a week

🞎 Once a week

🞎 Only on the weekends

🞎 Always

### **PART E – CES-D**

### Please indicate how often you had the following feelings **in the LAST WEEK:**

***Never or Sometimes Often Always or***

***almost never***  ***almost always***

***(<1 day) (1-2 days) (3-4 days) (5-7days***)

| F1. I was worried about things that usually don’t worry me | 🞏 | 🞏 | 🞏 | 🞏 |
| --- | --- | --- | --- | --- |
| F2. I didn’t feel like eating | 🞏 | 🞏 | 🞏 | 🞏 |
| F3. I couldn’t shake off my feelings of sadness, not even with the help of my friends and family | 🞏 | 🞏 | 🞏 | 🞏 |
| F4. I felt like I was as worthy as others | 🞏 | 🞏 | 🞏 | 🞏 |
| F5. It was hard for me to pay attention to what I was doing | 🞏 | 🞏 | 🞏 | 🞏 |
| F6. I felt depressed | 🞏 | 🞏 | 🞏 | 🞏 |
| F7. I felt like everything I did was difficult | 🞏 | 🞏 | 🞏 | 🞏 |
| F8. I had hope for the future | 🞏 | 🞏 | 🞏 | 🞏 |
| F9. I thought that my life was a failure | 🞏 | 🞏 | 🞏 | 🞏 |
| F10. I was afraid | 🞏 | 🞏 | 🞏 | 🞏 |
| F11. I had a restless sleep | 🞏 | 🞏 | 🞏 | 🞏 |
| F12. I was happy | 🞏 | 🞏 | 🞏 | 🞏 |
| F13. I spoke less than usual | 🞏 | 🞏 | 🞏 | 🞏 |
| F14. I felt lonely | 🞏 | 🞏 | 🞏 | 🞏 |
| F15. People weren’t friendly with me | 🞏 | 🞏 | 🞏 | 🞏 |
| F16. I had fun | 🞏 | 🞏 | 🞏 | 🞏 |
| F17. There were moments when I burst out crying | 🞏 | 🞏 | 🞏 | 🞏 |
| F18. I felt sad | 🞏 | 🞏 | 🞏 | 🞏 |
| F19. I felt like people didn’t like me | 🞏 | 🞏 | 🞏 | 🞏 |
| F20. I just couldn’t get going | 🞏 | 🞏 | 🞏 | 🞏 |

***THANK YOU FOR YOUR COOPERATION.***
